# Supplementary material for: Static posturography as a novel measure of the effects of aging on postural control in dogs
Source: PLoS One. 2022 Jul 8;17(7):e0268390. doi: 10.1371/journal.pone.0268390 (PMC9269968; doi:10.1371/journal.pone.0268390)
Supplement: S1 File — This file contains the physical, neurologic, and orthopedic examination sheets that were used to record clinical examination. (PDF) [file pone.0268390.s001.pdf]

THE LONGITUDINAL STUDY OF AGEING OF THE NERVOUS SYSTEM IN COMPANION DOGS

Patient label

Study ID:_________ Date:_____________ Visit month #:___________ Estimated Life stage:_____________

Physical Exam

Weight(kg): ___________ Temp: __________ Pulse: __________ Respiration: __________ MM/CRT: _________

Heart murmur (1-6): ________________________ BCS (1-9, see reference):_____________________

Body Systems

	Normal	Abnormal	Describe
EENT/oral			
CVR			
GIT/ABD			
MSK			
LYMPH			
DERM			
GU			
Ortho			

EXAMINATION NOTES:

____

____

UPDATES SINCE LAST VISIT

____

____

____

Neurologic exam

Patient Name:________________ Study ID:________ Date:_____________ Visit month #:________

1. Mental Status:_______________ Alert Depressed Stuporous Comatose
2. Posture:____________________ Normal - Head Tilt - Tremor - Falling - Paraparesis - Tetraparesis
3. Gait:_______________________ Shuffle/Waddle Ataxia Dysmetria Circling
4. Palpation: (0 = normal, 1 = mild, 2 = moderate, 3 = severe)

a.Neck pain: ___________ b.TL pain: ____________ c. LS pain: __________ d. Head pain________

5. Postural reactions: 0(absent), 1(reduced), 2(normal), 3(increased), 4(clonus)

	LF	RF	LH	RH
CP				
Hopping				

6. Cranial nerves: 0(absent), 1(reduced), 2(normal), 3(increased), 4(clonus)

	L	R
Menace (II, VII)		
Palpebral (V, VII)		
Oculovestubular reflex (III, VI, VIII)		
Gag reflex/Jaw tone (IX, X, V)		
Eye Position/Strabismus (III – VL, VI – medial)		
Facial muscles – eyelid, ear, lip, nose- (VII)		
Masticatory muscles – (V)		
Facial Sensation – (V)		
Nystagmus – (VIII)		

7. Spinal reflexes: 0(absent), 1(reduced), 2(normal), 3(increased), 4(clonus)

	LH			RH			LF	RF
Patellar reflex								
Cranial Tibial reflex								
Sciatic reflex								
Flexor Withdrawal	Hock	Stifle	Hip	Hock	Stifle	Hip		
Panniculus								
Deep Pain								
Perineal Reflex								

Gait/Orthopaedic exam

Patient label

Date: ________________

Gait:

____

____

		R Forelimb	L Forelimb	R Hindlimb	L Hindlimb
Posture	Standing (0-4)				
Walk and Trot	Lameness (0-5)				

Posture:
0: Normal
1: Slightly off-loading (favors limb but foot on floor; difficult to see)
2: Moderate offloading (favors limb but foot on floor; obvious)
3: Severe offloading (toe touching)
4: Not using limb at all.

Lameness:
0: No lameness
1: Intermittent and subtle lameness noted with the trained eye
2: Consistent mild weight-bearing lameness, typically with head bob
3: Significant weight-bearing lameness
4: Toe-touching lameness
5: Non-weight bearing lameness

Joint Evaluation

Patient label

Date:________________

Forelimb	ROM		Pain		Crepitus		Effusion		Thickening	
	L	R	L	R	L	R	L	R	L	R
Manus										
Carpus										
Elbow										
Shoulder										

Hindlimb	ROM		Pain		Crepitus		Effusion		Thickening	
	L	R	L	R	L	R	L	R	L	R
Pes										
Tarsus										
Stifle										
Hip										

Range of Motion:
0: normal; **1**: mild-moderate decreased; **2**: severely decreased

Pain based on manipulation:
0: Does not notice; **1**: Orients to site, does not resist or mild resistance (mild); **2**: Orients to site, slight objection to manipulation (moderate); **3**: Withdraws from manipulation, may vocalize, may turn to guard area (significant); **4**: Tries to escape/prevent manipulation, may bite or show aggression (severe)

Crepitus:
0: none, no crunching; **1**: mild, only feel crunching sometimes **2**: moderate, crunching felt always, may be painful;
3: severe, can feel and hear crunching, may be painful

Effusion:
0: none, no fluid pocket felt; **1**: mild, small fluid pocket felt only on palpation; **2**: moderate, prominent on palpation; **3**: severe, may see visible fluid pocket

Thickening:
0: none, can feel all anatomic structures easily; **1**: mild, less defined anatomic structures; **2**: moderate, can slightly define anatomic structures;
3: severe, can no longer feel anatomic structures
