# Supplementary material for: Static posturography as a novel measure of the effects of aging on postural control in dogs
Source: PLoS One. 2022 Jul 8;17(7):e0268390. doi: 10.1371/journal.pone.0268390 (PMC9269968; doi:10.1371/journal.pone.0268390)
Supplement: Attachment — Submitted filename: Static posturography as a novel measure of the effects of aging on postural control in dogs PLosONE review.docx [file pone.0268390.s003.docx]

Static posturography as a novel measure of the effects of aging on postural control in dogs.

This is a reasonably well written paper that generated a large volume of data. Interpretation of that data is challenging because the findings are not always uniform. The authors discussion of limitations and final conclusions are fair, but some clarifications are needed. My biggest obstacles with this paper were 1) interpreting how they selected data, 2) the shear volume of acryonyms and data presented will challenge many readers, 3) ICC is calculated on averaged data, not raw data and 4) the number of statistical evaluations performed.

67	“…cheaper…” this is brand dependent and certain brands are at least twice the cost of a force platform set up. Please remove.

91/442	Why 0.75 as a cutoff, why not the 0.7 cutoff as supported by this cited literature. Also, since time is a continuous variable, why not evaluate the data to see if there is change in COP associated with adjusted age. Regardless, at least provide rationale for making this cutoff. It may be as easy as this was exploratory research and other options beyond a 0.75 cutoff should be investigated.

98/409	Was this the extent of the morphometric measurements? Why dog height and not length and width? Not investigating if other morphometric measurements influence COP is a potential limitation of the study.

131	Is this the definition of a valid trial?

136	Do you mean 70 consecutive frames, from 7 trials? I’m unsure of what you did here given your previous description of inclusion criteria; were some dogs studied even though they didn’t achieve the “inclusion criteria”. Were these frames and trials randomly selected? In the discussion, you mention you averaged all of this data. Please clarify how you handled this data, it is unclear to me and I would not be able to reproduce this experiment. From the review of your data, it seems that you began with 5360 data points, from this you selected (random?)(and averaged?) 70 data points from 7 of 10 (random?) valid trials leaving your with 7 data points for each dog, then this was averaged. Is this what was statistically evaluated? Post hoc handling of your data needs to be clear and logical.

137	On line 129, you stated, “for inclusion, at least 10 valid trials were required”. Clarify how you could have data if “fewer than 7 individual trials were available”.

222	It seems this should be calculated on the raw data, not averaged data that is a subset of the raw data. Please defend this methodology or perform on raw data.

Tables	Often, the r^2 are significant, but quite small. Please address the importance of these findings.
